# Assessing the impact of TB/HIV services integration on TB treatment outcomes and their relevance in TB/HIV monitoring in Ghana

**DOI:** 10.1186/2049-9957-1-13

**Published:** 2012-12-24

**Authors:** Gloria Akosua Ansa, John D Walley, Kamran Siddiqi, Xiaolin Wei

**Affiliations:** 1 University Hospital, Legon, University of Ghana, P. O. Box LG 79, Legon, Accra, Ghana; 2 Nuffield Centre for International Health and Development, University of Leeds, 101 Clarendon Road, Leeds, LS2 9LJ, United Kingdom; 3 Department of Health Sciences, The University of York, Seebohm Rowntree Building, Heslington, York, YO10 5DD, United Kingdom; 4 School of Public Health and Primary Care, Chinese University of Hong Kong, Hong Kong, China

**Keywords:** Tuberculosis, HIV, Integration, Indicator, Treatment outcome, Referral, Partial integration, One-stop shop

## Abstract

**Background:**

The impact of the human immunodeficiency virus (HIV) on tuberculosis (TB), and the implications for TB and HIV control, is a public health challenge in Ghana – almost a quarter (23%) of all TB cases were HIV positive in 2010. The integration of TB/HIV services has therefore emerged as an essential component of the national response to TB and HIV. The aim is to reduce fragmentation, improve access, enhance efficiency and improve quality of care. Ghana’s TB/HIV policy comprises three linked sets of activities: effective implementation of the Stop TB Strategy for TB control, improved HIV prevention and care, and the implementation of additional TB/HIV activities. Different models of service delivery with increasing integration of TB/HIV activities are expected to provide greater access to more comprehensive care. The objective of this paper is to assess the impact of TB/HIV integration on TB treatment outcomes and to explore the usefulness of TB treatment outcomes as TB/HIV indicators.

**Methods:**

A before-and-after study to observe the introduction of TB/HIV activities into TB programmes in three hospitals with different levels of integration was conducted. Anonymised patient data was collated from TB registers from each facility, and analysed to determine if TB treatment outcomes changed significantly after integration.

**Results:**

TB treatment success was 50% (95% CI 49 – 52) prior to, and 69% (95% CI 65 – 73) after, integration (Χ^2^ 43.96, p < 0.00). Treatment success increased from 43% to 53% at the one-stop shop (OSS), from 69% to 78% at the partially integrated site (PIS) and substantially from 46% to 78% at the referral site (RS) (Χ^2^ 64.54; p<0.01). Defaults and cases transferred out reduced from 14.3% and 15.3% prior to integration, to 1.4% and 9.0% after integration, respectively, accounting for a significant increase in treatment success. Death rates remained high at 18% in all cases studied and 25% in HIV-associated cases after integration.

**Conclusion:**

TB/HIV integration may improve TB treatment success, but its exact impact is difficult to ascertain due to non-specificity and design limitations. TB mortality may be more useful as an indicator for monitoring TB/HIV activities in Ghana.

## Multilingual abstracts

Please see Additional file 1 for translations of the abstract into the six official working languages of the United Nations.

## Background

### Introduction

The impact of HIV on TB, and the implications for TB and HIV control, has been acknowledged as a public health challenge in Ghana, as is the case in many other Sub-Saharan African countries. Almost a quarter (23%) of all TB cases in Ghana were HIV positive in 2010 [1], up from 12% the previous year [2]. The WHO TB/HIV policy pursues the following objectives:

To establish and strengthen the mechanisms of collaboration and joint management between HIV programmes and TB-control programmes for delivering integrated TB and HIV services, preferably at the same time and location;

To reduce the burden of TB in people living with HIV and in their families and communities, and to initiate antiretroviral therapy (ART) early and in line with WHO guidelines; and

To reduce the burden of HIV in patients with presumptive and diagnosed TB, and in their families and communities, by providing HIV prevention, diagnosis and treatment [3].

TB treatment in Ghana dates back to the pre-independence era but the National TB Control Programme (NTP) was established in 1994 and the WHO-approved Directly Observed Treatment Short course (DOTS) strategy was then also adopted. TB control in Ghana is highly decentralised with services available in most health facilities and in some communities. National prevalence is estimated at 201 per 100,000 population [1]. The National AIDS and STIs Control Programme (NACP) was established in 1987, and ART was introduced on a pilot basis in 2003. HIV care and ART started with a very centralised approach, but have since been progressively decentralised from the teaching hospitals down to the district hospitals. Until 2007, TB and HIV services were mostly segregated and referral services were the only form of integration.

TB and HIV services were disconnected, which meant an increase in the cost of care for patients, as well as other added inconveniences, as numerous visits were required to access the required care. There were higher losses to follow-up and case fatalities, as well as delays in ART initiation. Although TB programme indicators like case notification, default rates and case evaluation had been progressively improving under the existing TB control interventions, death rates were high and treatment success remained below the global target of 85% [2]. In 2005, a national TB/HIV technical committee was set up to develop a TB/HIV policy and to define treatment guidelines, which were completed in 2007. In June 2007, TB/HIV activities were incorporated into the existing TB and HIV services.

The integration of TB and HIV services provides a unified strategy to address the burden of TB/HIV [4]. The aim of integrated health services is to organise and manage the services so that people can get the health care they need [5]. In TB and HIV control, integration of services has emerged as an essential component in any country’s response to the TB/HIV dual epidemic – the aim being to create coherence and synergy between the two programmes, not only to address problems with access and fragmentation, but also to enhance efficiency, quality of care and consumer satisfaction [6]. In line with this, the strategic framework of Ghana’s TB/HIV policy consists of three linked sets of activities: effective implementation of the Stop TB Strategy for TB control, improved HIV prevention and care, and the implementation of a set of additional collaborative TB/HIV activities [7].

### Conceptual framework

**Figure 1:**
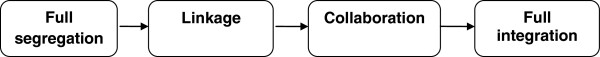
Continuum of integration of health services forming the basis of TB/HIV service delivery models.

Different models of service delivery for TB/HIV activities have been described [3,8] based on integration being a continuum from segregation (through linkage and collaboration) to full integration, according to the degree of integration [9-11]. In addition, increasing integration is expected to be associated with greater access to more comprehensive care (Figure 1).

This figure illustrates the continuum of integration from completely separated service units to full integration, where collaborating units combine to form a single unit. This model forms the basis for the service delivery models for TB/HIV services.

This improved access to comprehensive care is anticipated to improve the outcomes of both TB and HIV care [5,12]. Although three models have been previously described [4,7,8], the current WHO TB/HIV policy [3] expands on these to create a five-tier model (Table 1).

**Table 1:** Relationship between the level of integration and different service delivery models

**Level of integration**	**Study delivery models**	**WHO descriptions**
**Linkage**	Referral	Entry via TB service and referral for HIV testing and care.
		Entry via HIV service and referral for screening, diagnosis and treatment of TB.
**Collaboration**	Partially integrated	Entry via TB service and referral for HIV care after HIV testing.
		Entry via HIV service and referral after TB screening.
**Full Integration**	One-stop shop	TB and HIV services provided at a single facility.

TB treatment outcomes measured by the TB programme in Ghana include the following cases: cured, completed treatment, died, defaulted, failed or transferred out. The cases which are ‘cured’ or ‘completed treatment’ together constitute successful TB treatment outcomes, while the rest represent adverse or unsuccessful outcomes. TB treatment success is a measure of a TB programme’s capacity to retain patients throughout a complete course of treatment. It has a direct impact on mortality and is also influenced by other TB control processes [13]. In TB/HIV care, outcomes are useful for studying the performance of healthcare provision and for detecting problems in its implementation [13].

### Purpose of study

The national approach to the implementation of the TB/HIV policy has been gradual and incremental so that evidence of effective implementation can be used to inform nationwide scale-up. This paper is therefore part of a study that was intended to generate recommendations to inform TB/HIV integration. The specific objective of this paper is to assess the impact of TB/HIV integration on TB treatment outcomes and to explore the relevance of TB treatment outcomes as indicators of TB/HIV integration in Ghana. Findings from the comparisons of the effectiveness of the three different models will be reported in another paper.

## Methods

This study was conducted in the Eastern region of Ghana in three district hospitals, representing increasing levels of integration, namely referral site (RS), partially integrated site (PIS) and one-stop shop (OSS), which corresponded to linkages, collaboration and full integration, respectively (Figure 1, Tables 1 &2). The Eastern region consistently has one of the highest HIV prevalence in Ghana. At the time of the study, HIV prevalence was 4.2% in the region, with the highest rate being 5.8% in cities where the PIS and the RS were located. All three hospitals were located in urban areas and served as referral centres for health centres, community clinics and other private health facilities within the districts. The three main economic activities in the districts are agriculture, trading and the service industry.

**Table 2:** TB/HIV intervention delivery at TB treatment centres at study sites

**Characteristic**	**One-stop shop**	**Partial integration**	**Referral**
**HIV pre-test counselling**	Available	Available	Available
**HIV testing**	Available	Referral to HIV unit	Referral to HIV unit
**CPT initiation and/or continuation**	Available	Available	Referral to HIV unit
**ART initiation and/or continuation**	Available	Referral to HIV unit	Referral to HIV unit

A before-and-after study involving all TB patients registered between January 2006 and December 2008 was done at each of the sites. This was part of a PhD study data collection period. Data collection ended in September 2009 when all TB patients had completed their six- or eight-month treatment programmes.

This study was a sort of ‘natural experiment’ to observe introduction of collaborative TB/HIV activities into existing TB control activities for the first time and this happened in June 2007. Hence, January 2006 to May 2007 can be classified as ‘before integration’ and June 2007 to December 2008 as ‘after integration’. The TB/HIV intervention included providing recommended drug therapy for all registered TB cases, provider-initiated counselling and testing (PICT) of all registered TB cases for HIV, initiation of co-trimoxazole preventive therapy (CPT) to reduce other infections, and antiretroviral therapy (ART) for all eligible HIV-positive TB cases. There was also the introduction of a modified TB register that records TB/HIV activities.

Anonymised and aggregated patient data from TB registers of each facility were used to compute outcomes. The data was checked for accuracy, followed by descriptive and statistical analysis using Windows Excel and Statistical Product and Service Solutions (SPSS). ‘Cured’ and ‘completed’ outcomes were re-classified as ‘successful’, while the rest (died, defaulted, transferred and failure) were re-classified as ‘unsuccessful’. Missing data were not included in the determination of outcomes. Chi-square (X^2^) statistical tests were used to examine whether or not there was any association between TB/HIV integration and TB treatment outcomes. And probability (p) -values and confidence intervals were also employed to determine how significant differences in TB treatment outcomes before and after integration were. Ethical approval was obtained from the University of Leeds and the Ghana Health Service.

## Results

There were 1330 TB cases registered from January 2006 to December 2008: 727 (55%) were registered before integration and 603 (45%) were registered after. About 96% (1275) of these were evaluated at the end of treatment (Table 3).

**Table 3:** Demographic characteristics of evaluated cases (excluding cases for which outcomes were not declared)

	**Frequency (%)**				
	**Before (%)**	**After (%)**	**RS**	**PIS**	**OSS**
	**N = 708**	**N = 567**	**N = 616**	**N = 284**	**N = 375**
**Gender**					
**Female**	38.3	39.8	33.4	50.4	39.7
**Male**	61.7	60.2	66.6	49.6	60.3
**Age**					
**Mean (SD)**	40.5 (18.0)	41.8 (18.4)	41.8 (18.5)	38.0 (17.5)	42.2 (18.0)
**Median (IQR)**	40 (22)	39 (24)	41 (25)	37 (20)	40 (24)
**Range**	1 - 97	2 - 105	1 - 97	1 - 86	1 - 105
**Site of Disease**					
**Pulmonary**	92.7	88.5	88.3	90.1	95.9
**Extra-pulmonary**	7.3	11.5	11.7	9.9	4.5
**Sputum at month 0**					
**Positive**	49.3	51.7	51.3	42.9	53.7
**Negative**	32.2	47.0	31.4	46.4	44.6
**Not recorded**	18.5	1.3	17.3	10.7	1.7
**Patient Type**					
**New**	88.3	90.5	86.0	89.2	94.7
**Defaulter**	4.7	1.4	4.4	4.7	0.3
**Failure**	0.6	3.5	2.8	0.0	1.9
**Relapse**	5.6	4.1	5.7	5.8	2.9
**Transferred in**	0.7	0.5	1.0	0.4	0.3

**Legend:** SD - Standard deviation, IQR - Interquartile range.

A total of 708 (97%) cases registered before integration were evaluated at the end of TB treatment: 50% were successful, 19% died, 14% defaulted and 15% were transferred out after treatment had been initiated. After integration, 94% of cases were evaluated at the end of treatment: 69% were successful, 1% defaulted and cases transferred out reduced to 9%. Deaths remained high at 18% after integration (Table 4).

**Table 4:** TB treatment outcomes before and after integration at the study sites

	**Frequency (%)**							
	**All sites**		**RS**		**PIS**		**OSS**	
	**Before (%)**	**After (%)**	**Before (%)**	**After (%)**	**Before (%)**	**After (%)**	**Before (%)**	**After (%)**
**Cured**	17.8	27.0	18.7	28.7	22.1	30.6	11.9	22.6
**Completed**	32.6	42.0	27.1	49.8	47.2	45.4	30.7	30.2
**Defaulted**	14.3	1.4	22.8	0.8	0.0	0.0	9.7	3.0
**Died**	18.8	17.5	18.7	15.0	15.3	21.5	22.2	18.1
**Failure**	1.3	2.3	1.6	4.0	0.6	0.8	1.1	1.0
**Transferred out**	15.3	9.0	11.1	0.8	14.7	0.8	24.4	24.1
**Successful**	**50.4**	**69.0**	**45.8**	**78.5**	**69.3**	**76.0**	**42.6**	**52.8**
**95 % CI**	**48.8–52.4**	**64.9–72.9**	**43.9–48.2**	**70.8–86.2**	**61.9–76.7**	**65.7–86.3**	**39.9–45.3**	**48.9-56.6**

**Legend:** CI - confidence interval.

**Figure 2:**
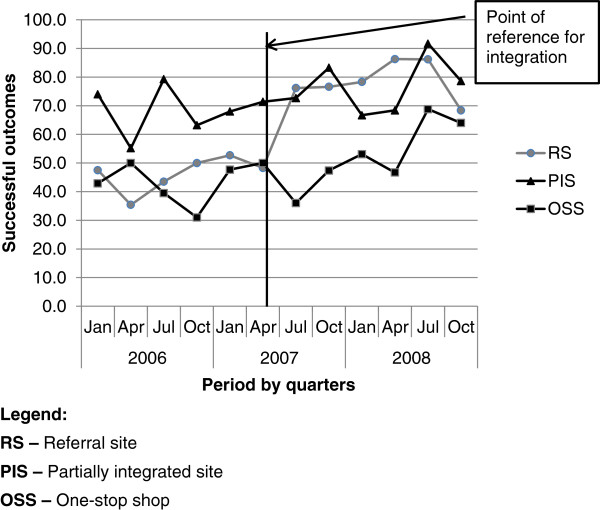
Trend of successful TB treatment outcomes at the three study sites over the study period.

Successful treatment outcomes seem to have increased significantly from 50% (95% CI 49–52) before, to 69% (95% CI 65–73) after integration (Χ^2^ 43.96, p < 0.00). There was no significant increase in treatment success after integration at the OSS (Χ^2^ 3.85, p<0.05) and PIS (Χ^2^ 1.56, p<0.26), but there appears to be a statistically significant increase after integration at the RS (Χ^2^ 64.54, p<0.00). Over the period of study, successful outcomes were lowest at the OSS (Figure 2). Although the PIS had highly successful results after integration, the change seemed significant only at the RS (Table 4).

This figure depicts the trend of quarterly successful TB treatment outcomes at the three study sites over the three-year period of study.

The results indicate that, in general, there may be a significant improvement in treatment success rates after integration and TB cases were most likely to have a successful outcome at the RS after integration.

Death rates reduced marginally from 18.8% (95% CI 18.5 - 19.0) before integration to 17.5% (95% CI 17.2 - 17.7) after, but this was not statistically significant. All three sites had high death rates with no notable reductions after integration, except at the PIS where the death rate increased from 15% to 22% (Table 4).

**Figure 3:**
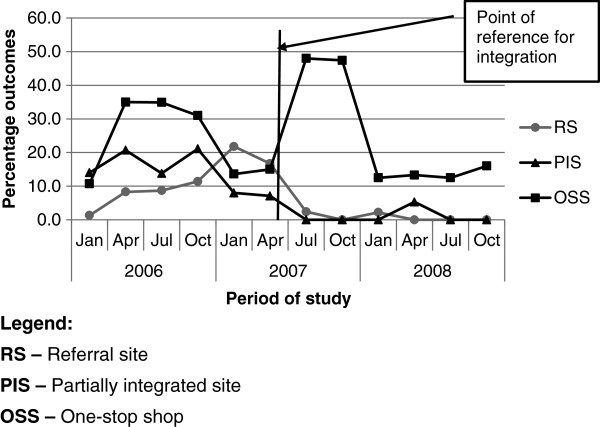
Trend of TB cases transferred out during treatment at study sites over the study period.

With cases transferred out during treatment, there was a marked difference between the OSS and the other two sites. The rates were generally higher at the OSS with peaks in 2006 and 2007 (Figure 3), and no significant difference after integration (Table 4). The other two sites had lower transfer rates and demonstrated a further reduction after integration (Figure 3, Table 4).

This is a figure of the trend of the quarterly percentages of TB patients transferred out at each of the three study sites over the period of study. Cases transferred out represent one of the key adverse outcomes of TB treatment.

Defaulter rates fell significantly from 14.3% (95% CI 14.1 - 14.4) to 1% after integration (Χ2 66.55, p < 0.00). At the individual sites, defaults reduced most significantly at the RS, but were more unstable at the OSS. There was, however, no record of default at the PIS, which suggests problems with recording or signals an inaccurate classification of cases.

Treatment success was 72% (95% CI 66–78) in HIV-negative TB cases, as compared to 64% (95% CI 59–69) in HIV-positive cases, while mortality in HIV-positive cases was 25% (95% CI 24–26), as compared with 9.8% (95% CI 9.7 - 10.0) among negative cases. Among HIV-positive TB cases, treatment success was 75% for those who were receiving ART and 61% for those who were not. Mortality was 23% for those who were receiving ART and 26% for those who were not.

## Discussion

The results of this study suggest that, after integration, successful TB treatment outcomes may have increased, but this apparent increase is largely due to the notable increase that was observed only at the RS. The increase was also lower than national levels due to factors such as high mortality, particularly among HIV-positive patients [14,15]. TB/HIV integration is primarily about changing the processes of care and multidisciplinary collaboration [16], and it is anticipated to increase patient-centeredness, enhance coordination and improve continuity [6]. TB treatment outcomes would subsequently improve through better access, better resource utilisation and enhanced efficiency [6,17]. However, other concurrent TB or HIV, specific interventions may lead to improved outcomes as well. Therefore, when TB/HIV integration is introduced as part of a national programme-improvement approach, successful TB treatment outcomes are then a representation of the impact of all the interventions. The specific impact of each individual intervention becomes difficult to assess. This underscores their non-specificity [18] as TB/HIV indicators. In this study, for example, improvement in TB treatment success was mainly due to decreases in ‘default’ and ‘transferred out’ cases, which may have only improved as a result of other TB-specific control interventions that were in place, such as community-based TB treatment and the use of treatment supporters in all three study sites [19,20]. Consequently, although integration has the potential to improve TB treatment outcomes, the extent of its contribution may be challenging to assess due to the impact of concurrent strategies.

Among the adverse treatment outcomes, death rates were high at all sites and reduced only marginally after integration. Even though TB deaths include deaths from any cause whilst undertaking TB treatment, research has established that HIV-associated TB is related to an increased risk of TB deaths [21]. Elliot et al. [22] demonstrated that most deaths in cases of HIV-associated TB result from active TB and its complications, or complications of the HIV infection itself. TB case fatality rates in Africa are 16-35% in HIV-positive patients not receiving ART and 4-9% in HIV-negative patients [15]. The study concurs with the study undertaken for this research paper as mortalities in HIV-positive TB patients were 25%, accounting for 59% of all TB deaths, as compared to 10% among HIV-negative cases, accounting for 25% of all deaths. Even though the treatment success of 75% among HIV-positive TB cases receiving ART after integration was comparable to the 72% as was demonstrated by Huerga et al. [23], death rates were much higher at 23% in HIV-associated cases, as opposed to the 10% in HIV-negative cases.

Based on the high death rates in the HIV-associated TB cases with little or no apparent influence by other TB-control processes, this study therefore suggests that TB/HIV integration may have a more direct impact on TB deaths than on successful treatment outcomes. While HIV-associated TB accounted for 59% of all TB deaths, the other concurrent TB-control interventions seem to have had much less impact on mortality in Ghana as compared to their impact on defaults and transfers out, which significantly contributed to improved TB treatment success. TB deaths may thus be a more sensitive indicator for TB/HIV integration i.e. TB deaths among TB patients, including those with HIV as well, may provide useful information on the impact of TB/HIV integration.

It is recommended that TB deaths be further explored as an indicator to monitor the impact of TB/HIV integration in similar contexts. This is with reference to the propositions of Maher et al. [24] that TB deaths are crucial for monitoring programme performance, but are limited by incomplete coverage of all incident TB cases, inaccurate routine programme reporting of deaths, and the unknown contribution of deaths from TB and HIV alone. Even though Maher et al’s study focused on countries with high HIV prevalence in addition to heavy TB burden, this study also suggests that TB mortalities can be useful for monitoring TB/HIV integration in low-income countries with relatively higher TB burden but lower HIV prevalence. More research into the causes of death among HIV-positive TB patients is therefore required to identify and understand the causes of death in order to properly assess its real usefulness as a TB/HIV indicator.

Lack of randomisation and controls limit the internal validity of this study as it subjects it to temporal and selection bias. For that reason, the findings of this study are presented not as categorical statements on the impact of integration, but as a piloting of methods. More rigorous studies are needed to assess the exact impact of TB/HIV integration, and to improve TB/HIV monitoring and evaluation. Another limitation is that evaluation was mainly based on TB-related indicators due to the different programme information systems, and the challenges associated with access to HIV-patient data at the time of the study.

## Conclusions

In conclusion, this study suggests that although TB/HIV integration has the potential to improve TB treatment outcomes, assessing the exact contribution that TB/HIV integration can have is challenging and requires more rigorous evaluative studies. Due to the high TB death rates among HIV-associated cases, TB mortality may be a more useful indicator to use when researching the TB/HIV burden in Ghana.

## Abbreviations

ART: Antiretroviral therapy; CI: Confidence interval; CPT: Co-trimoxazole preventive therapy; DOTS: Directly observed therapy short course; HIV: Human Immunodeficiency Virus; OSS: One-stop shop; PICT: Provider-initiated counselling and testing; PIS: Partially integrated site; RS: Referral site; SPSS: Statistical product for service solutions; Stop TB: WHO Stop TB strategy; TB: Tuberculosis.

## Competing interests

The authors declare that there are no competing interests.

## Authors’ contributions

GAA conceived the study, designed it, acquired the data and analysed it, and drafted the manuscript. JDW, KS and XW participated in the design of the study, the data analysis and the revision of the manuscript for important intellectual content. All authors read and approved the final manuscript.

## Supplementary Material

Additional file 1 Multilingual abstracts in the six official working languages of the United Nations. Click here for file
