# Supplementary material for: Assessing the impact of TB/HIV services integration on TB treatment outcomes and their relevance in TB/HIV monitoring in Ghana
Source: Infect Dis Poverty. 2012 Dec 24;1:13. doi: 10.1186/2049-9957-1-13 (PMC3710204; doi:10.1186/2049-9957-1-13)
Supplement: Additional file 1 — Multilingual abstracts in the six official working languages of the United Nations. [file 2049-9957-1-13-S1.pdf]

Tanslation of the abstract into the six official working languages of the United Nations

مرض السل / فيروس نقص المناعة البشرية "الأيدز" في غانا

غلوريا أكوسوا أنسا، وجون دي. والي، وكامران صديقي، وشياو لين وى

ملخص

F h: يعتبر تأثير فيروس نقص المناعة البشرية "الأيدز" (HIV) على مرض السل(TB) ، فضلاً عن الآثار المترتبة على مكافحة السل والأيدز، تحديًا يواجه الصحة العامة في غانا – حيث أظهر نحو ربع (23٪) من جميع حالات السل أنهم مصابين بفيروس الأيدز في عام 2010. وقد برز دمج خدمات مكافحة السل / الأيدز (TB/HIV) بالتالي كعنصر أساسي للاستجابة الوطنية في مواجهة السل والأيدز. والهدف من ذلك هو الحد من التشرذم وتحسين الوصول إلى المرضى وتعزيز الكفاءة وتحسين جودة الرعاية. وتضم سياسة غانا في مواجهة السل / الأيدز ثلاث مجموعات مرتبطة من الأنشطة: التنفيذ الفعال للاستراتيجية دحر السل (Stop TB) لمكافحة مرض السل، وتحسين الوقاية من الأيدز ورعاية المرضى المصابين به، وتنفيذ أنشطة إضافية ذات علاقة بالسل / الأيدز. ومن المتوقع أن توفِر النماذج المختلفة لتقديم الخدمات مع زيادة دمج أنشطة السل / الأيدز قدرًا أكبر من الوصول إلى الرعاية الأكثر شمولاً. والهدف من هذه الورقة هو تقييم أثر دمج خدمات مكافحة السل / الأيدز على نتائج علاج السل واستكشاف جدوى نتائج علاج السل كمؤشرات على حجم الإصابات بالسل / الأيدز.

ǚ: تم إجراء دراسة قبل وبعد الدمج لمراقبة إدخال أنشطة السل / الأيدز إلى برامج مكافحة السل في ثلاثة مستشفيات بمستويات مختلفة من الدمج. وتم جمع بيانات المرضى المجهولين من سجلات مرضى السل من كل مرفق، وتحليلها لتحديد ما إذا كانت نتائج علاج السل قد تغيرت بشكل ملحوظ بعد الدمج.

YiǚǨǚ: كان النجاح في علاج السل بنسبة 50٪ (95٪ CI 49 - 52) قبل الدمج (X^2 43.96، P < 0.00)، و بنسبة 69٪ (9595٪ CI 65 - 73) بعده. زاد نجاح العلاج من 43٪ إلى 53٪ في الموقع المتكامل (OSS)، من 69٪ إلى 78٪ في موقع متكامل جزئيًا (PIS) وبشكل كبير من 46٪ إلى 78٪ في موقع الإحالة (RS) (X^2 64.54، P < 0.01). وقد انخفضت الغيابات وحالات نقل من 14.3٪ من 15.3٪ قبل الدمج، إلى 1.4٪ و9.0 ٪ بعد الدمج، على التوالي، وهو ما يمثل زيادة كبيرة في نجاح العلاج. ظلت معدلات الوفيات عالية بنسبة 18٪ في جميع الحالات التي تمت دراستها و25٪ في الحالات المرتبطة بالأيدز بعد الدمج.

F Əǚ: قد يُحسّن دمج خدمات السل / الأيدز من نجاح علاج السل، ولكن من الصعب تأكيد تأثيره الدقيق بسبب قيود عدم التخصص والتصميم. وقد تكون وفيات السل أكثر فائدة كمؤشر لرصد أنشطة السل / الأيدز في غانا.

Translated from English version into Arabic by Heba Salah, through

TRANSLATORS
WITHOUT BORDERS

评估结核病/艾滋病毒综合性防治服务对结核病的疗效及其对加纳结核病/艾滋病毒监测的相关影响

Gloria Akosua Ansa, John D. Walley, Kamran Siddiqi, Xiaolin Wei

摘要

引言：在加纳，艾滋病毒（HIV）对结核病（TB）的影响，以及 TB 和 HIV 防治可能产生的影响是一个公共卫生挑战。在 2010 年，23%的 TB 病例 HIV 检测为阳性。因此，TB/HIV 综合性防治服务成为国家应对 TB 和 HIV 的一个重要组成部分。其目的是减少片段化，提高可及性，提高工作效率和护理质量。加纳的 TB/HIV 防治政策包括 3 个相互联系的部分：有效实施遏制 TB 的防治策略，提高对 HIV 预防与关怀，实施额外的 TB/HIV 防治活动。随着 TB/HIV 防治服务日益一体化，不同模式的服务交付将提供更多的机会以获得更加全面的照料。本研究的目的是评估 TB/HIV 综合性防治服务对 TB 治疗效果的影响，并探讨 TB 疗效作为 TB/HIV 双重感染的指标的效用。

方法：在 3 个医院观察不同程度的 TB/HIV 综合性防治服务加入 TB 防治计划之前和之后的效果。从 TB 记录表中收集不记名患者的数据，并作分析，以确定实施综合性防治服务后 TB 疗效是否发生了显著变化。

结果：在实施 TB/HIV 综合性防治服务前，TB 治疗成功率为 50% (95% CI 49 – 52)，实施后为 69% (95% CI 65 – 73) (X^2 43.96, $p < 0.00$)。一站式服务（OSS）将治疗成功率从 43%提高到 53%，在部分整合（PIS）的情况下，治疗成功率从 69%升至 78%，在参照点（RS）治疗成功率则从 46%提高至 78% (X^2 64.54; $p<0.01$)。中断治疗（defaulted）和转出（transferred out）病例分别从实施综合性防治服务前的 14.3%、15.3%降至 1.4%、9.0%，并且治疗成功率也大幅提高。在实施综合性防治服务后所有研究病例的死亡率仍高达 18%，同时为 HIV 感染者中的死亡率为 25%。

结论：TB/HIV 综合性防治服务可提高结核病的治疗成功率，但由于非特异因素和设计上的限制，难以确定该防治服务的确切影响。在加纳，结核病死亡率可能是 TB/HIV 防治活动更有用的监测指标。

Translated from English version into Chinese by Yang Pin, through

CHINESE CENTER FOR DISEASE CONTROL AND PREVENTION
NATIONAL INSTITUTE FOR PARASITIC DISEASES

Évaluer l'incidence de l'intégration des services TB/VIH sur les résultats de traitement de la tuberculose et leur pertinence en matière de suivi de la TB/du VIH au Ghana

Gloria Akosua Ansa, John D. Walley, Kamran Siddiqi, Xiaolin Wei

Résumé

Informations de fond : L'incidence du virus de l'immunodéficience humaine (VIH) sur la tuberculose (TB) et les implications en matière de lutte contre la tuberculose et de contrôle du VIH constituent un problème de santé publique au Ghana - près d'un quart (23%) de l'ensemble des cas de tuberculose étaient en effet séropositifs au VIH en 2010. L'intégration des services TB/VIH est donc apparue comme un élément essentiel de la réponse nationale à apporter au problème de la tuberculose et du VIH. L'objectif est de réduire la fragmentation, d'améliorer l'accès, d'accroître l'efficacité et d'améliorer la qualité des soins. La politique du Ghana en matière de TB/VIH comprend trois volets : la mise en œuvre effective de la stratégie Halte à la tuberculose de lutte antituberculeuse, l'amélioration de la prévention du VIH et des soins, et la mise en oeuvre de nouvelles activités TB/VIHs. Il existent différents modèles de prestation de services qui intègrent de plus en plus des activités relatives à la tuberculose/au VIH censées permettre un accès plus étendu à l'ensemble des services de soins. Cet article a pour objet d'évaluer l'impact de l'intégration des services TB/VIH sur les résultats de traitement de la TB et d'explorer l'utilité de ces résultats en tant qu'indicateurs TB/VIH.

Méthodes : Une étude « avant-après » a été menée afin d'observer l'introduction d'activités liées à la TB/au VIH à des programmes de lutte contre la tuberculose dans trois hôpitaux et à différents niveaux d'intégration. Des données de patients anonymes ont été recueillies auprès des registres TB de chaque établissement et analysés pour déterminer si les résultats de traiteemnt de la tuberculose avaient sensiblement changés après l'intégration des activités.

Résultats : Le traitement de la tuberculose était couronné de succès dans 50 % des cas avant (IC à 95% 49 - 52)et dans 69% des cas après (IC à 95% 65 - 73) l'intégration des activités (X^2 43,96 , p < 0,00). Le succès du traitement a augmenté, passant de 43% à 53% au *one-stop shop* (OSS), de 69% à 78% au site partiellement intégré (PIS) et de 46% à 78%, soit une augmentation considérable, au site de référence (RS) (X^2 64,54 ; p<0,01). Les cas faisant défaut et cas transférés hors de l'établissement ont diminués de 14,3 % et 15,3 % avant l'intégration, et sont tombés respectivement à 1,4 % et 9,0 % après l'intégration, résultant ainsi en une augmentation significative de réussite du traitement. Le taux de décès est resté élevé à 18% dans tous les cas étudiés et à 25% dans les cas associés aux VIH après intégration.

Conclusion : L'intégration de services de TB/VIH peut améliorer l'issue du traitement de la TB bien que son incidence exacte soit difficile à déterminer en raison de la non-spécificité et des limites de conception. La mortalité due à la TB peut être plus utile en tant qu'indicateur de suivi des activités TB/VIH au Ghana.

Translated from English version into French by Lola Cello-Kirkham, through

TRANSLATORS
WITHOUT BORDERS

Влияние интеграции услуг, направленных на борьбу с туберкулезом/ВИЧ, на лечение туберкулеза, а также оценка важности этих услуг в качестве составной части программы контроля заболеваемости туберкулезом/ВИЧ в Гане.

Глория Акосуа Анса, Джон Д. Уэлли, Камран Сиддики, Сиаолин Вей

Краткий обзор

Предпосылки: Воздействие вируса иммунодефицита человека (ВИЧ) на заболеваемость туберкулезом (ТБ) и устранение факторов, негативно влияющих на контроль заболеваемости ТБ/ВИЧ, являются основными задачами, стоящими перед общественным здравоохранением Ганы. Это связано с тем, что в 2010 году около четверти (23%) случаев заболевания туберкулезом было зарегистрировано у ВИЧ-положительных пациентов. В связи с этим, оказание услуг, направленных на борьбу с ТБ/ВИЧ, рассматривается в качестве необходимого компонента программы снижения количества больных этими заболеваниями. Основной целью вышеупомянутых услуг является улучшение доступности, эффективности и качества медицинского обслуживания в стране. Политика Ганы, направленная на борьбу с ТБ/ВИЧ, предусматривает принятие трех видов мер, непосредственно связанных между собой: эффективное внедрение стратегии по контролю и устранению заболеваемости туберкулезом, улучшенная профилактика ВИЧ и более эффективное лечение ВИЧ-положительных пациентов, а также внедрение дополнительных мер по борьбе с ТБ/ВИЧ. Ожидается, что различные формы предоставления услуг с более эффективной схемой интеграции программ по борьбе с ТБ/ВИЧ сделают комплексное медицинское обслуживание более доступным. Целью данной работы является оценка влияния интеграции услуг, направленных на борьбу с ТБ/ВИЧ, на эффективность лечения ТБ, а также изучение применимости результатов лечения ТБ в качестве показателей ТБ/ВИЧ.

Методы: Для наблюдения за процессом внедрения мер по борьбе с ТБ/ВИЧ в программы лечения ТБ в трех медицинских учреждениях с различной степенью интеграции вышеупомянутых услуг проводился анализ "До и после". Из архива туберкулезного отделения каждого из этих учреждений были получены анонимные данные пациентов, которые подвергались последующему анализу с целью определения степени воздействия интеграции услуг на эффективность лечения больных ТБ.

Результаты:Эффективность лечения ТБ составляла 50% (доверительный интервал 95%: 49-52) до начала интеграции мер и 69% (доверительный интервал 95%: 65-73) после (X^2 43,96, $p < 0,00$). Процент случаев успешного излечения увеличился с 43% до 53% в

комплексных медицинских учреждениях, с 69% до 78% - в медицинских учреждениях с частичной интеграцией и с 46% до 78% - в медицинских учреждениях, направляющих своих пациентов в учреждения с комплексным обслуживанием (X^2 64,54; $p<0.01$). Количество переданных случаев и случаев несоблюдения медицинских предписаний снизилось с 14,3% и 15,3% соответственно до начала интеграции до 1,4% и 9,0% после. Кроме того, значительно повысилась эффективность лечения. Показатель смертности после интеграции мер остался на высоком уровне: 18% всех рассмотренных случаев и 25% пациентов с ВИЧ.

Заключение:Интеграция мер, направленных на борьбу с ТБ и ВИЧ, способна повысить эффективность лечения ТБ, однако точно оценить ее влияние не представляется возможным по причине ее универсальности и наличия ограничений. Показатель смертности больных ТБ может использоваться для более точной оценки эффективности мер по борьбе с ТБ на фоне ВИЧ в Гане.

Translated from English version into Russian by Tatyana Kovaleva Modesto, through

TRANSLATORS
WITHOUT BORDERS

Efectos del tratamiento integrado contra la tuberculosis y el VIH y su relevancia en el control de la tuberculosis y el VIH en Ghana

Gloria Akosua Ansa, John D. Walley, Kamran Siddiqi y Xiaolin Wei

Resumen

Antecedentes: Los efectos del virus de la inmunodeficiencia humana (VIH) sobre la tuberculosis (TB) y su repercusión en el control de dichas enfermedades son un grave problema sanitario en Ghana, donde casi un cuarto (23 %) de los enfermos de TB también eran portadores del VIH en 2010. Por tanto, la integración de ambos servicios se ha convertido en una parte fundamental de la respuesta nacional ante la coinfección. Los objetivos son reducir la fragmentación del tratamiento, mejorar su eficacia y calidad, y facilitar el acceso al mismo. La política sanitaria del país consta de tres conjuntos de actividades relacionadas: aplicar la estrategia «Alto a la tuberculosis» para controlar la enfermedad, mejorar el tratamiento y la prevención del VIH, y seguir llevando a cabo nuevas acciones contra la TB y el VIH. Con este nuevo modelo de prestación de servicios cada vez más integrados se espera poder ofrecer un mejor tratamiento a un mayor número de población. El objetivo de este artículo es analizar el efecto de la integración de ambos servicios sobre los resultados en el tratamiento de la TB y explorar la utilidad de dichos resultados como indicadores de TB y VIH.

Métodos: Se ha realizado un estudio previo posterior para evaluar la introducción de acciones contra la TB y el VIH en los programas de control de la TB de tres hospitales con distinto grado de integración de servicios. Se recopilaron los datos anónimos de los pacientes de TB para determinar si los resultados del tratamiento variaban de forma significativa tras la integración.

Resultados: La tasa de éxito del tratamiento de la TB fue del 50 % (intervalo de confianza del 95 % entre 49 y 52) antes de la integración (X2 43.96; $p < 0.00$), y del 69 % (intervalo de confianza del 95 % entre 65 y 73) después de esta. El éxito aumentó del 43 % al 53 % en el centro integrado, del 69 % al 78 % en el centro parcialmente integrado y, de forma muy significativa, del 46 % al 78 % en el centro de remisión (X2 64.54; $p < 0.01$). Los casos remitidos y no atendidos antes de la integración se redujeron del 15.3 % y el 14.3 % al 9.0 % y 1.4 % después de esta, lo que representa un aumento importante de la tasa de éxito. Las tasas de mortalidad se mantuvieron en el 18 % en todos los casos, y en el 25 % de los casos asociados al VIH después de la integración.

Conclusión: El tratamiento integrado de la TB y el VIH puede mejorar los resultados del tratamiento de la TB, pero es difícil asegurar cuánto debido a las limitaciones de diseño de este

estudio y su carácter general. Es posible que la tasa de mortalidad de la TB sea un mejor indicador para el seguimiento de la TB y el VIH en Ghana.

Translated from English version into Spanish by Rosa Sanz, through

TRANSLATORS
WITHOUT BORDERS
